# Characterization of different types of anxiety disorders in relation to structural integration of personality and adverse and protective childhood experiences in psychotherapy outpatients – a cross-sectional study

**DOI:** 10.1186/s12888-023-04988-2

**Published:** 2023-07-12

**Authors:** Jonathan Nowak, Christoph Nikendei, Ivo Rollmann, Maximilian Orth, Hans-Christoph Friederich, David Kindermann

**Affiliations:** Centre for Psychosocial Medicine, Department of General Internal Medicine and Psychosomatics, University of Heidelberg, Thibautstraße 4, 69115 Heidelberg, Germany

**Keywords:** Generalized anxiety disorder, Panic disorder, Phobia, Structural integration of personality, Adverse childhood experiences, Protective childhood experiences

## Abstract

**Background:**

Current research has emphasized the role of structural integration of personality and childhood experiences for the understanding of anxiety disorders. In this study, we examined the relationship between anxiety disorders (generalized anxiety disorder vs. panic disorder vs. phobic disorders), the level of structural integration of personality, and negative and protective childhood experiences at the beginning of outpatient psychodynamic psychotherapy treatment. Differences were characterized in comparison to patients with no anxiety disorders.

**Methods:**

The sample included a total of 1646 outpatient psychodynamic psychotherapy treatments, of which 695 treatments included the diagnosis of at least one anxiety disorder. Levels of structural integration of personality were assessed according to the Operationalized Psychodynamic Diagnosis (OPD-2) system. Self-reported negative and protective childhood experiences were examined by using the Questionnaire for the Assessment of Adverse and Protective Childhood Experiences (APC). Associations were tested using single factor ANOVAs.

**Results:**

Patients with anxiety disorders showed lower levels of structural integration of personality and reported more adverse childhood experiences than patients with no anxiety disorders. Regarding the subscales of structural integration of personality, phobic disorders were associated with impaired external communication, whereas for generalized anxiety disorder, an (uncorrected) association with impaired self-regulation was found. Also, generalized anxiety disorder was associated with sexual abuse and other traumatization (accidents etc.) during childhood, while panic disorder and phobic disorders were associated with emotional neglect, abuse, and fewer protective childhood experiences.

**Conclusions:**

Our findings emphasize the need of considering structural integration of personality and childhood experiences in order to understand and treat various types of anxiety disorders.

**Supplementary Information:**

The online version contains supplementary material available at 10.1186/s12888-023-04988-2.

## Background

Anxiety disorders, including phobias, panic disorder, and generalized anxiety disorder, show a lifetime prevalence of up to 29% in international retrospective and prospective studies [1–4]. These prevalence rates make anxiety disorders one of the most common mental disorders. While prevalence rates in past decades have been estimated as remaining stable [5], a global raise in prevalence of about 25% in the first year of the COVID-19 pandemic has been suggested by a recent WHO scientific statement based on the Global Burden of Disease 2020 study [6, 7]. Furthermore, taking into account the association of anxiety disorders with enhanced health care utilization [8], elevated disability-adjusted life years [9], chronification and recurrence [10], financial burden to the health care system [11], social stigmatization [12], and commonly delayed recognition and treatment efforts [13, 14], a profound understanding of anxiety related psychopathology is of significant importance.

Within the current versions of the Diagnostic and Statistical Manual of Mental Disorders (DSM-5) [15] and the International Classification of Diseases (ICD-11) [16], anxiety disorders are subclassified into generalized anxiety disorder, panic disorder, agoraphobia, specific phobia, social anxiety disorder, separation anxiety disorder, and selective mutism. According to the DSM-5 and ICD-11 definitions, generalized anxiety disorder is defined as persisting anxiety with excessive apprehension (‘free-floating anxiety’) and worry about everyday activities and events [15, 16]. Panic disorder is defined as repeated panic attacks that are unexpected and not restricted to specific situations [15, 16]. Agoraphobia is defined as occurring in situations wherein escape or help might be unavailable (e.g., public transport, open or enclosed spaces, crowds), whereas social anxiety disorder is defined as occurring in social situations and interactions (e.g., conversations, being observed when eating/drinking or holding a speech) [15, 16].Additionally, specific phobia occurs in response to specific objects, environments or situations (e.g., animals, flying, heights, blood, injections) [15, 16]. Typical comorbidities of anxiety disorders include major depressive disorder, bipolar disorder, substance use disorder, obsessive-compulsive disorder, post-traumatic stress disorder, and personality disorders [17, 18]. The latest research has intensively discussed the relationship between anxiety disorders and personality functioning [19].

The concept of personality structure [20] reflects a dimensional assessment of personality functioning based on psychodynamic theory. Structural integration of personality is, for instance, a prominent part of the Operationalized Psychodynamic Diagnosis (OPD-2) system [21, 22]. The OPD Levels of Structural Integration Axis (OPD-LSIA) [23] consists of four functional domains with eight dimensions (self-perception and object perception, self-regulation and regulation of relationships, internal communication and external communication, attachment to internal objects and attachment to external objects), each of which are further subdivided into three subdimensions (e.g., affect tolerance, impulse control, and regulation of self-esteem as subdimensions of self-regulation). Conceptual similarities with the OPD-LSIA can be found, for instance, in the Levels of Personality Functioning Scale (LPFS) dimensions from the Alternative DSM-5 Model for Personality Disorders [15, 24] and in the concept of emotional intelligence [25].

Within the framework of the OPD-LSIA, deficits in structural integration of personality may be caused by adverse childhood experiences and early childhood traumatization, showing high prevalence rates (40–50%) in epidemiological studies ([26–28]. Adverse childhood experiences are defined as experiences of “abuse and household dysfunction during childhood” [26], including the categories of psychological abuse (e.g., insulting or frightening behavior of parents), physical abuse (e.g. hitting, pushing, slapping, potentially including marks and injuries), and sexual abuse (e.g., touching in a sexual way, attempt of sexual intercourse), as well as household exposure to domestic violence, substance use, mental illness and criminal behavior. As shown in an extensive amount of research, adverse childhood experiences are known as important risk factor not only for the development of anxiety disorders, but also for a large spectrum of other mental (e.g., substance use, violence to self and others, sexual risk behavior, major depressive disorder, suicide attempts) and somatic (e.g., obesity, diabetes, cancer, heart disease, respiratory disease) health consequences during adulthood [29]. The influence of positive childhood experiences, including protective (indirect effect by compensating negative experiences) and promotive (direct effect on childhood development) factors, has been suggested as being a possible resilience factor against the detrimental effects of adverse childhood experiences [30].

Recent research has emphasized a general relationship between anxiety disorders and impaired personality functioning [19]. For instance, an impairment of self-directed and interpersonal functioning has been suggested to be relevant for several subtypes of anxiety disorders [31, 32]. However, other studies did not find a relationship between anxiety disorders and impairment of personality functioning [33, 34]. It is unclear to what extent different subtypes of anxiety disorders might be affected by impaired personality functioning and which specific subdomains of personality dysfunction might be relevant. While it was claimed that impaired personality functioning was present in all subtypes of anxiety disorders regardless of the specific type of anxiety disorder [32], strong severity of interpersonal disturbances was suggested in particular for generalized anxiety disorder [35]. Furthermore, theoretical concepts have suggested a link between different types of anxiety disorders and different levels of structural integration of personality [36]. Similarly, a general association between anxiety disorders and adverse childhood experiences has been reported in an extensive body of research [37–39]. In particular, generalized anxiety disorder has frequently been associated with severe forms of negative childhood experiences, such as sexual and physical abuse, separation experience, and a dysfunctional family situation [40, 41]. Also, generalized anxiety disorder has been linked more often to the general occurrence of adverse childhood experiences than panic disorder [42]. Nevertheless, there is still a lack of studies directly comparing specific subtypes of anxiety disorders with regard to distinct categories of child maltreatment and stressful early life events. Therefore, it remains unclear to what extent specific forms of adverse childhood experience might be connected to specific anxiety disorder types. Moreover, the occurrence of protective childhood experiences among patients with anxiety disorders has not been investigated thus far.

The aim of the present study was to examine the relationship between anxiety disorders according to DSM-IV criteria and (1) differential patterns of structural integration of personality according to the Operational Psychodynamic Diagnosis system (OPD-2) [21]; and (2) self-reported adverse and protective childhood experiences at the beginning of outpatient psychotherapeutic treatment. We hypothesized that (a) anxiety disorders would be associated with differences in level of structural integration of personality and self-reported adverse and protective childhood experiences compared to treatment cases with no anxiety disorders, and that (b) following theoretical conceptions on anxiety disorders and structural integration of personality [36], different types of anxiety disorders (i.e., generalized anxiety disorder vs. panic disorder vs. phobic disorders) would be associated with differences in level of structural integration, as well as (c) differences in self-reported adverse and protective childhood experiences.

## Methods

### Participants

Our sample consisted of *n* = 1646 outpatient psychotherapeutic treatments (64.4% female, age *M* = 34.8, *SD* = 13.1 years, range 18–85 years) that were conducted at the outpatient training clinic for psychodynamic therapy at the University Hospital Heidelberg in Germany [43] between January 2013 and July 2021. Patients had to be sufficiently proficient in German or English and had to participate in at least one preparatory session with a therapist to be included in the study. According to the fourth edition of the Diagnostic and Statistical Manual of Mental Disorders (DSM-IV) [44, 45], major depressive disorder was present in 79.2% of the treatments, while criteria for an anxiety disorder were fulfilled in 46.5% of the cases. In 20.0% of the treatments, at least one comorbid personality disorder was present. In 13.3% of the cases, criteria of a comorbid substance use disorder were fulfilled. In 24.2% of the cases, psychopharmacological medication was present in the patients’ medical history, but not at the beginning of the treatment sessions. However, in 21.7% of the treatments, psychopharmacological medication was still present at the beginning of psychotherapy.

### Procedure

In order to clarify the indication for psychotherapy, patients attended a clinical intake interview session before the beginning of treatment. After being informed accordingly, patients gave written informed consent to participate in the study. Patients were then asked to provide sociodemographic information and to complete several psychometric instruments. At this point, prior to the first preparatory session with a study therapist, the questionnaires relevant to the present study (Operationalized Psychodynamic Diagnosis Structure Questionnaire, OPD-SQ; Questionnaire for the Assessment of Adverse and Protective Childhood Experiences, APC) were assessed. Next, patients participated in a standardized diagnostic interview session (SCID-I and SCID-II) [46, 47] conducted by trained graduate students (at least B.Sc. in psychology). Afterwards, the first preparatory session with a randomly assigned study therapist took place.

### Ethics

The study protocol was developed according to the Helsinki II declaration [48]. Prior to recruitment of patients and therapists, the study was approved by the independent ethics committee of the Medical Faculty of the Heidelberg University (S-195/2014).

### Instruments

The ***Operationalized Psychodynamic Diagnosis Structure Questionnaire (OPD-SQ)*** is a self-assessment tool to evaluate the level of structural integration of personality as conceptualized by axis IV of the OPD-2 [21, 49]. Higher scores represent a lower level of structural integration of personality. The questionnaire contains 95 items and measures eight subscales with a five-point Likert scale from ‘0 = not true at all’ to ‘4 = completely true’. Patients were asked to answer the OPD-SQ after the clinical intake interview. Internal consistency was high for perception/cognition of the self (α = 0.89) and objects (α = 0.85), regulation of the self (α = 0.85) and relationships (α = 0.85), communication with the internal (α = 0.78) and external world (α = 0.74), and attachment to internal (α = 0.81) and external objects (α = 0.77).

The ***Questionnaire for the Assessment of Adverse and Protective Childhood Experiences (APC)*** [50] is a self-assessment questionnaire which assesses two scales: protective childhood experiences (α = 0.95) and adverse childhood experiences (α = 0.93). Adverse childhood experiences can further be divided into emotional neglect and abuse (α = 0.92), physical neglect and abuse (α = 0.55), sexual abuse (α = 0.89), traumatic experiences (α = 0.51), separation experiences (α = 0.63), dysfunctional family situation (α = 0.77), and missing or dysfunctional peer-group experiences (α = 0.57). Patients were asked to answer the APC after the clinical intake interview. Items were scaled on a five-point Likert scale from ‘0 = never’ to ‘4 = very often’. As each subscale of the short version only consists of two items, we could not calculate a Cronbach’s alpha. Thus, we followed the authors recommendation and calculated the Cronbach’s alpha for the long version. Nevertheless, we used the short version for all other steps during data analysis, as it shows better reliability than the long version [51].

### Data analysis

**Fig. 1:**
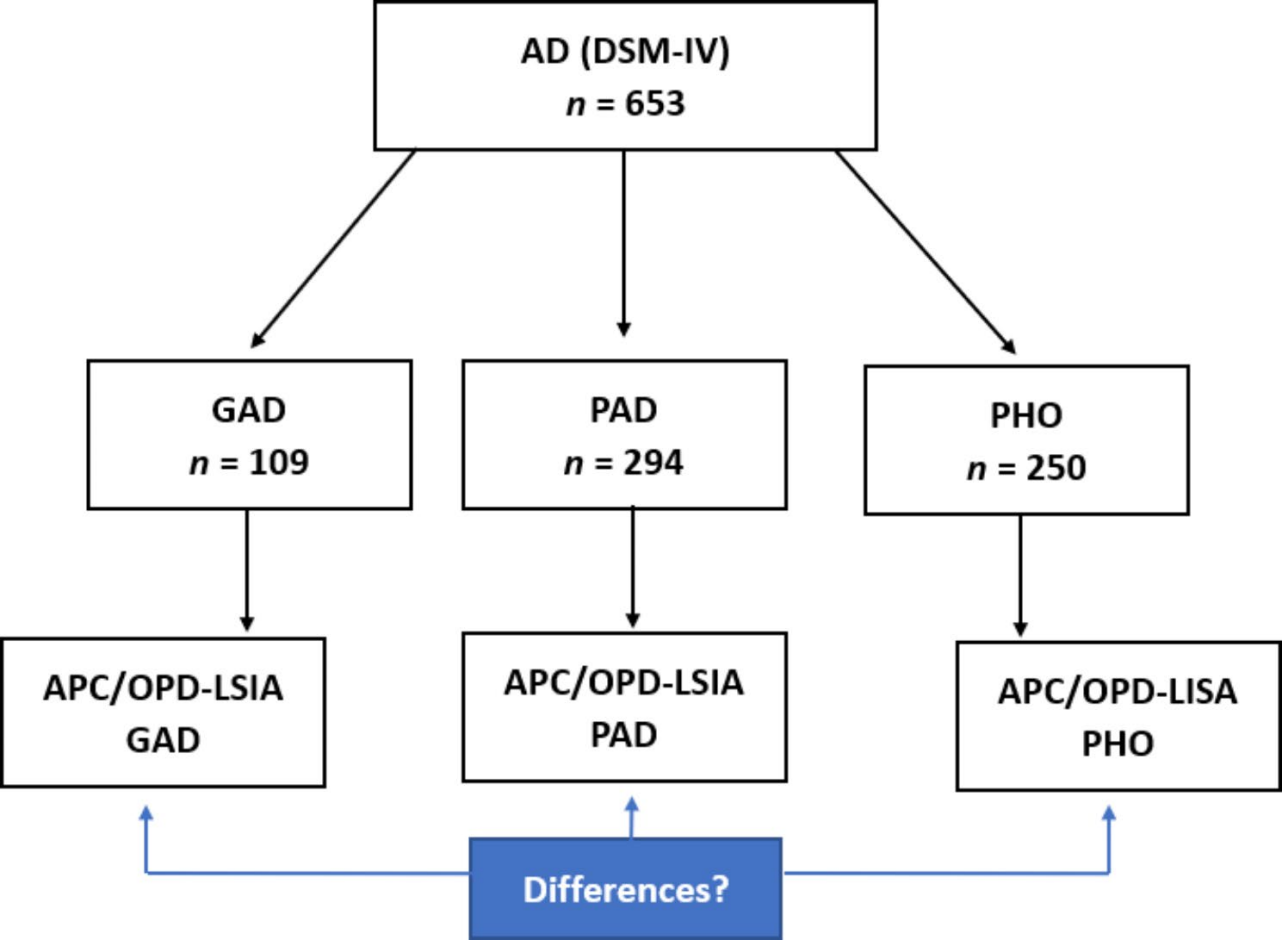
Subgroups of Treatment Cases involving Anxiety Disorders Note. AD = anxiety disorders. DSM-IV = Diagnostic and Statistical Manual of Mental Disorders, Fourth Edition. GAD = generalized anxiety disorder. PAD = panic disorder (± agoraphobia, but no GAD). PHO = phobic disorders (including isolated agoraphobia, social anxiety disorder, specific phobias, but no GAD / no PAD). APC = Questionnaire for the Assessment of Adverse and Protective Childhood Experiences. OPD-LSIA = Operationalized Psychodynamic Diagnosis System, Level of Structural Integration Axis

All analyses were calculated using R environment [52]. The aim of the study was to conduct group comparisons between patients with and without anxiety disorders, as well as on the subgroup level between different types of anxiety disorders. For this purpose, patients were divided based on their SCID-I diagnoses into a total of 4 groups: patients with generalized anxiety disorder, patients with panic disorder but no generalized anxiety disorder, and patients with phobic disorders but no panic disorder or generalised anxiety disorder (see also Fig. 1). The fourth comparison group consisted of the remaining patients who did not fullfill the criteria for an anxiety disorder according to DSM-IV. We compared these four groups concerning their sociodemographic variables, subscales of the OPD-SQ and the subscales of the APC. For these comparisons, we conducted single factor ANOVAs followed by Tukey-Kramer post-hoc tests for unequal sample sizes.

### Potential influence of the COVID-19 pandemic on sample characteristics

Since the period of data assessment included the first year of the COVD-19 pandemic, the characteristics of the sample before and after the onset of the pandemic were analysed and are described in more detail in a table in the additional material (Additional Table 1). As the date of pandemic onset, March 22 2020 was chosen, as this date represents the day of the first nationwide ‘lockdown’ measures initiated by the German Federal Government [53]. For this purpose, patients whose clinical intake interviews were conducted before March 22 2020 were included in the “pre-pandemic” group, while patients whose intake interviews took place after March 22 2020 were included in the “pandemic” group. Group comparisons showed that central characterstics of the sample did not differ substantially before and after pandemic onset. In both subsamples, mean age was about 33–35 years with about 64–68% female gender. About 74–80% of patients in both groups were diagnosed with a depressive disorder and about 20–22% of patients in both groups still took psychopharmacological medication at the beginning of treatment. Also, differences in the main scales of the APC (adverse and protective childhood experiences) and the OPD-SQ (structural integration of personality) remained marginal after pandemic onset. However, the number of diagnosed anxiety disorders (49.0% in the pre-pandemic group vs. 31.2% in the pandemic group), personality disorders (21.5% in the pre-pandemic group vs. 10.7% in the pandemic group) and substance disorders (14.9% in the pre-pandemic group vs. 3.3% in the pandemic group) decreased after pandemic onset, as did the number of patients with psychopharmacological medication in the past medical history (25.8% in the pre-pandemic group vs. 15.6% in the pandemic group). Since the subsamples did not substantially differ in the relevant target variables (childhood experiences, structural integration of personality), we decided to conduct our analyses for the total sample.

**Table 1:** Differences in Percentage of Sociodemographic Data

	GAD	Panic	Phobia	NoAD
Female	77.01%	69.51%	64.56%	61.01%
Unemployed	23.53%	24.90%	38.83%	26.03%
In Relationship	67.44%	57.02%	43.96%	49.73%
Current Psychotropic Drugs	35.96%	37.55%	25.71%	23.32%
Past Psychotropic Drugs	27.78%	43.55%	34.45%	24.87%

Note. GAD = generalized anxiety disorder. Panic = panic disorder. NoAD = no anxiety disorders

### Missing data

We calculated Little’s MCAR with the null hypothesis that missing data are completely randomly distributed [54]. The test was not significant (p > .99). We could therefore assume that removing the missing data would not affect our analysis [55]. Therefore, missing values were removed from our analysis.

### Transparency and openness

We report how we determined our sample size, all data exclusions, all manipulations, and all measures in the study, and we follow the “Journal Article Reporting Standards” (JARS) [56], the “Strengthening the Reporting of Observational Studies in Epidemiology” (STROBE) guidelines [57], and the “Statistical Analyses and Methods in the Published Literature” (SAMPL) guidelines [58]. The analysis code is available at: 10.11588/data/W63LJJ.

Data cannot be shared due to restrictions by the ethical review board. Data were analyzed using R, Version 4.1.3 [52]. The study design and data analysis were not pre-registered.

## Results

### Prevalence of anxiety disorders in the examined sample

Within our sample of 1646 outpatient treatments, 109 cases met DSM-IV criteria for generalized anxiety disorder, 294 fulfilled the criteria for panic disorder but no generalized anxiety disorder, and 250 fulfilled criteria for a phobic disorder but no panic disorder or generalized anxiety disorder. For the remaining treatments (*n* = 993), no criteria for an anxiety disorder were met.

### Sociodemographic data

As seen in Table 1, the socio-demographic analysis showed that the proportion of women differed between the subgroups, being the lowest for patients with no anxiety disorders and the highest for patients with generalized anxiety disorder. Unemployment was highest among patients with phobic disorders. Patients with generalized anxiety disorder reported the highest number of intimate relationships. Patients with panic disorder showed the highest frequency of psychopharmacological medication before or at the beginning of therapy.

As can be seen in Table 2, patients with phobic disorders had the lowest average age, and, accordingly, the lowest number of children. On average, patients with panic disorder had received more psychotherapeutic and inpatient psychiatric treatments before the start of current outpatient psychotherapy than the other three groups. Between the subgroups, differences in average numbers of DSM IV diagnoses were seen: while patients with no anxiety disorder showed an average of 1.62 diagnoses, patients with a phobic disorder or panic disorder showed an average of 3.44 diagnoses. Patients with generalized anxiety disorder showed an average of 4.39 diagnoses.

**Table 2:** Significant Differences in Sociodemographic Data

	GAD		Panic		Phobia		NoAD		*p*
	M	SE	M	SE	M	SE	M	SE	
Age	35.90	1.25	34.41	0.81	31.11	0.81	35.83	0.49	**< 0.01**
Children	0.84	0.13	0.67	0.07	0.49	0.06	0.71	0.04	**0.021**
Psychotherapies	0.54	0.08	0.81	0.06	0.66	0.06	0.46	0.03	**< 0.01**
Psychiatry	0.23	0.06	0.57	0.05	0.27	0.04	0.26	0.02	**< 0.01**
Diagnoses	4.39	0.17	3.44	0.10	3.44	0.09	1.62	0.04	**< 0.01**

Note. GAD = generalized anxiety disorder. Panic = panic disorder. NoAD = no anxiety disorders. Psychotherapies = number of previous psychotherapies. Psychiatry = number of previous psychiatric inpatient treatments. Diagnoses = number of SCID diagnoses. Significant *p*-values are highlighted

### Structural integration of personality

**Fig. 2:**
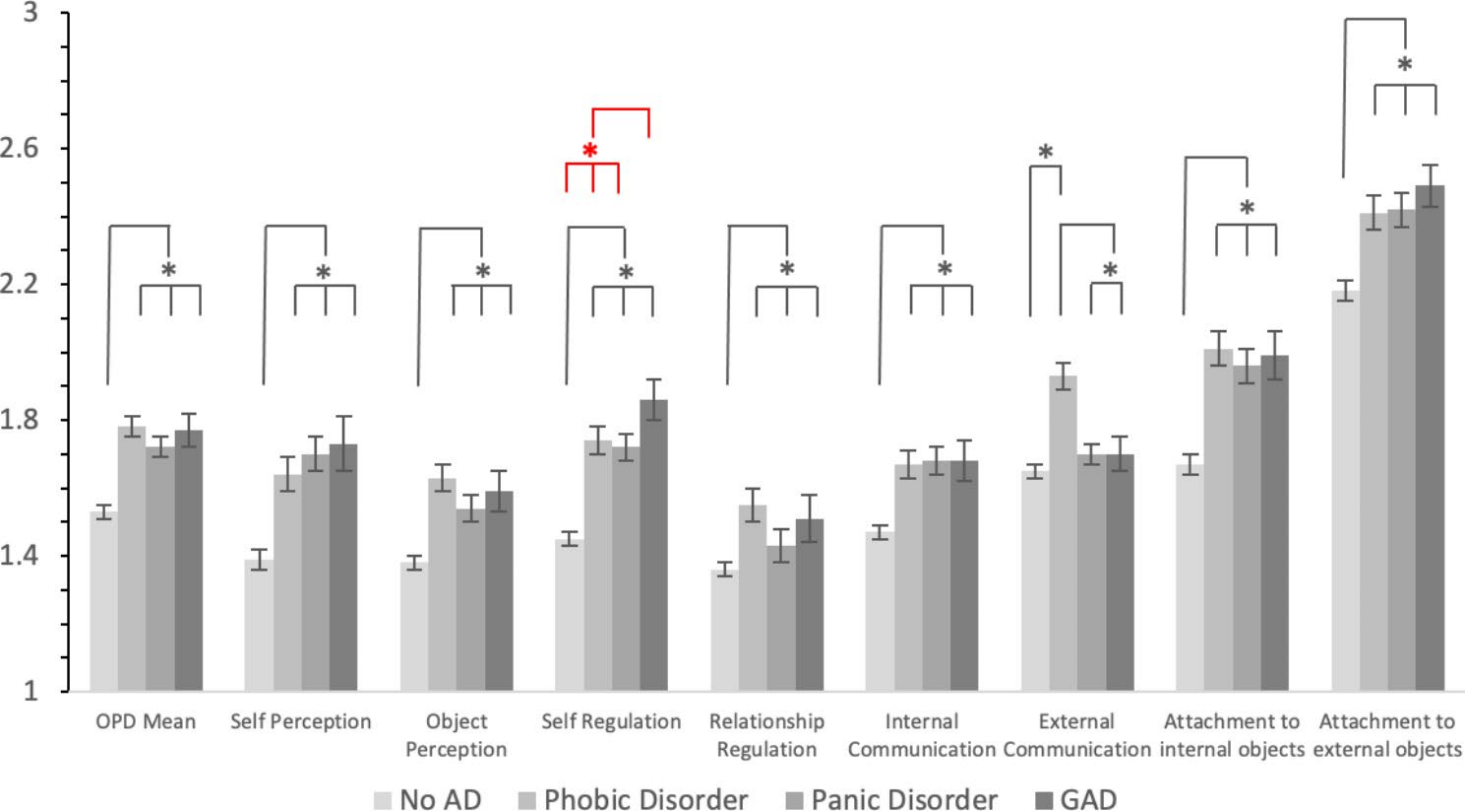
Differences in Structural Integration of Personality between Anxiety disorders Note. For better readability we started the y-axis with 1. NoAD = no anxiety disorders. GAD = generalized anxiety disorder. Error bars indicate standard errors. *p < .05. Markings in red: Not significant after post-hoc correction

There were significant differences in structural integration of personality on the main scale and all subscales of the OPD-SQ (Table 3; Fig. 2). With the exception of external communication, patients with no anxiety disorders reported significantly higher structural integration than all other groups. For external communication, patients with phobic disorders showed lower structural integration than all three other groups. For self-regulation, patients with generalized anxiety disorder showed lower structural integration than all three other groups in the uncorrected group comparisons. However, this difference was no longer present after post-hoc correction.

**Table 3:** Differences in Structural Integration between Anxiety Disorders

	GAD		Panic		Phobia		NoAD		*p*
	M	SE	M	SE	M	SE	M	SE	
OPD Mean	1.77	0.05	1.72	0.03	1.78	0.03	1.53	0.02	**< 0.01**
Self Perc.	1.73	0.08	1.70	0.05	1.64	0.05	1.39	0.03	**< 0.01**
Object Perc.	1.59	0.06	1.54	0.04	1.63	0.04	1.38	0.02	**< 0.01**
Self Reg.	1.86	0.06	1.72	0.04	1.74	0.04	1.45	0.02	**< 0.01**
Relation Reg.	1.51	0.07	1.43	0.05	1.55	0.05	1.36	0.02	**< 0.01**
Internal Comm.	1.68	0.06	1.68	0.04	1.67	0.04	1.47	0.02	**< 0.01**
External Comm.	1.70	0.05	1.70	0.03	1.93	0.04	1.65	0.02	**< 0.01**
Attachment IO	1.99	0.07	1.96	0.05	2.01	0.05	1.67	0.03	**< 0.01**
Attachment EO	2.49	0.06	2.42	0.05	2.41	0.05	2.18	0.03	**< 0.01**

Note. GAD = generalized anxiety disorder, Panic = panic disorder, NoAD = no anxiety disorders, OPD = Operationalized Psychodynamic Diagnosis System. Perc. = perception, Reg. = regulation, Comm. = communication, IO = internal objects, EO = external objects. Significant *p*-values are highlighted

### Adverse and protective childhood experiences

**Fig. 3:**
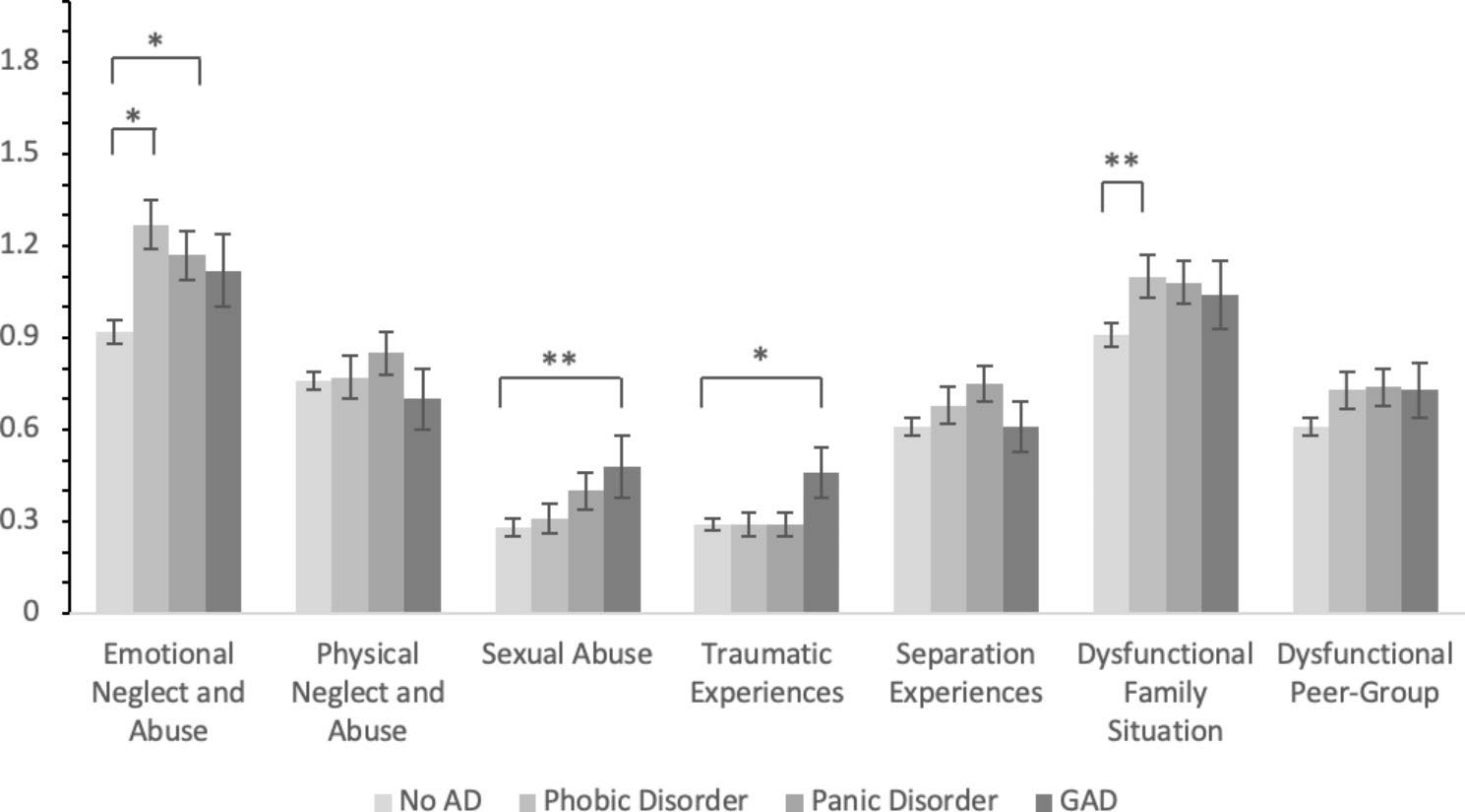
Differences in Subscales of the APC between Anxiety Disorders Note. APC = Questionnaire for the Assessment of Adverse and Protective Childhood Experiences. NoAD = no anxiety disorders. GAD = generalized anxiety disorder. Error bars indicate standard errors. *p < .05, **p < .01

Our analyses of variance revealed significant differences in protective and adverse childhood experience between the four groups (Table 4). Post-hoc tests revealed that patients with no anxiety disorders reported significantly more protective childhood experiences than patients with phobic disorders and panic disorder. Although patients with generalized anxiety disorders reported almost as many protective childhood experiences as patients with no anxiety disorders, the difference with phobic disorders and panic disorder was not significant. Furthermore, patients with no anxiety disorders reported significantly fewer adverse childhood experiences than all other three groups. The post-hoc comparisons of the APC subscales (Fig. 3) showed that patients with phobic disorders and panic disorder reported more emotional neglect and abuse than patients with no anxiety disorders. Also, patients with phobic disorders reported more dysfunctional family situations than patients with no anxiety disorders. In contrast, patients with generalized anxiety disorders reported more sexual abuse than patients with no anxiety disorders and also more other traumatic experiences (e.g. accidents) than patients with no anxiety disorders.

**Table 4:** Differences in Childhood Experiences between Anxiety Disorders

	GAD		Panic		Phobia		NoAD		*p*
	M	SE	M	SE	M	SE	M	SE	
Protective CE	2.57	0.09	2.38	0.06	2.37	0.06	2.61	0.03	**< 0.01**
Adverse CE	0.85	0.06	0.91	0.04	0.92	0.04	0.75	0.02	**< 0.01**
Emot. NaA	1.12	0.12	1.17	0.08	1.27	0.08	0.92	0.04	**< 0.01**
Phys. NaA	0.70	0.10	0.85	0.07	0.77	0.07	0.76	0.03	0.549
Sexual Abuse	0.48	0.10	0.40	0.06	0.31	0.05	0.28	0.03	**0.027**
Traumatic Exp.	0.46	0.08	0.29	0.04	0.29	0.04	0.29	0.02	0.075
Separation Exp.	0.61	0.08	0.75	0.06	0.68	0.06	0.61	0.03	0.150
Dysf. Family	1.04	0.11	1.08	0.07	1.10	0.07	0.91	0.04	**0.034**
Dysf. Peer-G.	0.73	0.09	0.74	0.06	0.73	0.06	0.61	0.03	0.083

Note. GAD = generalized anxiety disorder. Panic = panic disorder. NoAD = no anxiety disorders. CE = childhood experience, Emot. NaA = emotional neglect and abuse, Phys. NaA = physical neglect and abuse, Exp. = experience, Dysf. Family = dysfunctional family situation, Dysf. Peer-G. = dysfunctional peer-group situation. Significant *p*-values are highlighted

## Discussion

The aim of our study was to assess structural integration of personality and self-reported adverse and protective childhood experiences among 1646 treatment cases with and without anxiety disorders at the beginning of outpatient psychotherapy. In particular, we sought to investigate differences in structural integration of personality and childhood experiences within different types of anxiety disorders (generalized anxiety disorder, panic disorder, and phobic disorders) compared to patients with no anxiety disorders.

Regarding the level of structural integration of personality, patients with anxiety disorders showed lower levels than patients with no anxiety disorders in the overall mean of the OPD level of structural integration axis as well as in most subscales. Furthermore, patients with phobic disorders showed significantly lower integration in the domain of external communication (making contact, communicating affect, empathy) than patients with generalized anxiety disorder, patients with panic disorder, and patients with no anxiety disorders. For the uncorrected group comparisons, patients with generalized anxiety disorder also showed lower structural integration in the domain of self-regulation (subdomains: affect tolerance, impulse control, regulation of self-esteem) than patients with panic disorder, patients with phobic disorders, and patients with no anxiety disorders, although these differences were not significant when applying post-hoc correction.

The concept of structural integration of personality is a specific approach of assessing personality functioning [23]. Therfore, our results can be seen in line with recent findings on impaired personality functioning associated with anxiety disorders [19]. For instance, impaired personality functioning was found to be present in all subtypes of anxiety disorders – namely generalized anxiety disorder, panic disorder, and phobic disorders – compared to healthy controls when using the Structured Interview for Personality Organization (STIPO) [32, 59] for assessing personality functioning. Similarly, lower levels of self-differentiation according to the Differentiation of Self Inventory (DSI-R) [60], and higher levels of avoidant attachment according to the Experiences of Close Relationships Questionnaire (ECR-R) [61], were found in patients with anxiety disorders when compared to healthy controls [31]. Furthermore, correlations between anxiety symptoms and aspects of impaired personality functioning have been reported [62–64]. Interestingly, impaired personality functioning as a result of comorbid personality disorders has also been linked to treatment-resistance of anxiety disorders [65]. Hereby, patients seem to profit from intensified treatment taking into account aspects of personality functioning [65]. However, other studies could not confirm a relationship between anxiety disorders and impaired personality functioning [33, 34].

Regarding our findings of impaired external communication in patients with phobic disorders and the (uncorrected) findings of impaired self-regulation in patients with generalized anxiety disorder, our results are in contrast to a previous study [32], in which no differences in level of personality functioning *between* different anxiety disorder subtypes (generalized anxiety disorder vs. panic disorder vs. phobic disorders) were found. One reason for the lack of specific findings in this previous study [32] may lie in the relatively small sample size (patients with anxiety disorders: *n* = 97), whereas the larger size of our sample (treatments involving anxiety disorders: *n* = 653) made it statistically more likely to detect specific differences in structural integration of personality among different anxiety disorder subtypes. Also, the sample in the earlier study [32] included many patients with comorbid personality disorders (*n* = 63, two thirds of anxiety disorder patients), whereas our sample included a noticeably smaller proportion of comorbid SCID-II diagnoses among patients with anxiety disorders (*n* = 175; 26.8%). Overall, our results may converge with theoretical conceptions of different levels of structural integration of personality underlying different anxiety disorder types, especially a lower level of structural integration associated with generalized anxiety disorder, as opposed to other types of anxiety disorders [36].

Taken together, our results not only support previous findings of anxiety disorders being associated with impaired structural integration of personality, but also shed light on potential differences in structural integration of personality between distinct anxiety disorder types (i.e., impaired external communication in patients with phobic disorders, impaired self-regulation in patients with generalized anxiety disorder). The latter has not yet been shown in previous studies, presumably since our study includes larger samples sizes than most previous research [19]. Nonetheless, the fact that large sample sizes seem to be necessary to detect these differences implies that statistical effects are rather small and were certainly difficult to detect by previous studies due to lack of statistical power. Still, as our subgroup of cases with generalized anxiety disorder (*n* = 109) was presumably still underpowered, one future research question could be if differences in structural integration of personality are detectable to a greater extent when larger subgroups of patients with generalized anxiety disorder are involved in group comparisons. Another research question may lie in longitudinal aspects, e.g. the influence of treatment on impaired structural integration of personality in patients with anxiety disorders in the course of psychotherapy, especially with regard to different subtypes of anxiety disorders.

Regarding adverse and protective childhood experiences, patients with no anxiety disorders and patients with generalized anxiety disorders reported significantly more positive childhood experiences than patients with phobic disorders and patients with panic disorder. In contrast, patients with anxiety disorders reported significantly more adverse childhood experiences than patients with no anxiety disorders. Concerning subtypes of adverse childhood experiences, patients with generalized anxiety disorders reported significantly more experiences of sexual violence and of other traumatization (e.g., accidents, catastrophic events) than patients with no anxiety disorders. Also, patients with phobic disorders and patients with panic disorder reported more experiences of emotional neglect and abuse than patients with no anxiety disorders. Additionally, patients with phobic disorders reported more experiences of dysfunctional family situations than patients with no anxiety disorders.

Our results are in line with extensive previous findings on the general association between anxiety disorders and childhood traumatization [29, 37–39, 66–68]. With respect to differences in self-reported adverse childhood experience alongside different subtypes of anxiety disorders, our findings of patients with generalized anxiety disorder reporting higher frequencies of sexual abuse and other traumatization (accidents, etc.) than patients with no anxiety disorders are also consistent with previous results on the relationship between stressful childhood events and the likelihood of generalized anxiety disorder onset in later life. For instance, generalized anxiety disorder has been associated with sexual abuse, but also physical abuse, neglect, parental history of mental disorders, death of a parent, and divorce of parents in a large national comorbidity survey [40]. Also, generalized anxiety disorder has generally been associated more often with stressful childhood events than panic disorder, whereas for both disorder types, parental history of mental disorders has been identified as a shared risk factor [42]. Furthermore, taking into account reported associations between generalized anxiety disorder and childhood separation events, parental overprotection and dysfunctional family situations [41], generalized anxiety disorder seems to represent the anxiety disorder subtype with the strongest link to childhood traumatization and adverse childhood experiences. This finding might also be reflected by the respective findings in our study, where some of the more extreme forms of childhood traumatization (sexual abuse, other traumatization) were reported more frequently by patients with generalized anxiety disorder compared to the group with no anxiety disorders, while patients with panic disorder and phobic disorders more often reported other forms of interpersonal adverse experiences (emotional neglect and abuse, dysfunctional family situation) compared to patients with no anxiety disorders.

Altogether, our findings not only add evidence to previous results of anxiety disorders being strongly connected to adverse childhood experiences, but also support the notion that different types of anxiety disorders may be associated with different kinds of negative childhood experiences. Specifically, we found generalized anxiety disorder to be connected to some of the more extreme forms of adverse childhood experiences (sexual abuse, catastrophic events), while interpersonal and family-related forms of negative childhood events (emotional abuse and neglect, dysfunctional family situation) were especially associated with phobic disorders and panic disorder. To our knowledge, such differential aspects of adverse childhood experiences in relation to different anxiety disorder types have so far not been investigated by direct group comparisons in a sample of this size. Interestingly, these results are at least to some extent mirrored by our findings regarding structural integration of personality, wherein phobic disorders were associated with impaired external communication and generalized anxiety disorder (though uncorrected) with impaired self-regulation. Future research should therefore address potential interrelations between adverse childhood experiences and specific impairment of structural integration of personality within different types of anxiety disorders. In particular, potential links between impaired external communication (e.g. communicating affect) and interpersonal and family-related negative childhood events among patients with phobic disorders should be addressed. Furthermore, links between impaired self-regulation (e.g. affect tolerance) and severe childhood traumatization among patients with generalized anxiety disorder are of great relevance for future research. Similarly, exploring the influence of different kinds of adverse childhood experiences on treatment outcome during psychotherapy of different types of anxiety disorders might be a valuable future research goal.

With regard to self-reported protective childhood experiences, our results of fewer protective experiences in the groups with panic disorder and phobic disorders seem to mirror our findings of a surplus of adverse experiences in the group with anxiety disorders compared with the group with no anxiety disorders. This potential interplay might, at least partly, be explained by the lack of buffering effects of protective childhood experiences against the detrimental influence of negative events in these groups. However, the design of the applied APC questionnaire does not allow differentiation between protective and promotive childhood experiences, of which the latter are supposed to more directly exhibit positive effects on developmental trajectories during childhood [30]. One exception to the described pattern is the (uncorrected) higher amount of self-reported protective experiences among generalized anxiety disorder patients compared to patients with panic disorder and phobic disorders. One hypothetical explanation for this finding might lie in a tendency among generalized anxiety disorder patients towards idealization of positive past experiences and ‘splitting’ between positive and negative experiences, which have both been conceptualized as a central feature of disorders with lower levels of structural integration of personality [69].

Considering that the influence of positive childhood experiences on the development of anxiety disorders has, to our knowledge, not been investigated so far, further research is required in this field. Potential research questions addressing positive childhood experiences should include the differential influence of direct (promotive) and indirect (protective) factors in relation to structural integration of personality within different types of anxiety disorders. Another important research question could lie in the influence of positive childhood experience on treatment outcome in the course of psychotherapy of anxiety disorders.

Regarding the reduced number of diagnosed anxiety disorders in our sample alongside the COVID-19 pandemic compared to the pre-pandemic period, several explanations could account for this finding. As social distancing alongside lockdown and quarantine measures has been associated with increased anxiety symptoms including health anxiety in the general population [70], patients with pre-existing anxiety disorders may have experienced elevated levels of health anxiety related avoiding behavior, preventing them from making use of psychotherapy in the outside world, as it would mean leaving their safe environment at home. Social distancing and stay-at-home orders during the pandemic may also have led anxiety patients to prefer other, less risky treatment options, such as the use of psychopharmaceuticals, over the riskier (in terms of viral exposure) option of visiting a clinic to request a psychotherapy program. In addition, higher levels of anxiety symptomatology in the general population during the pandemic [70–72] may have led to greater social acceptance of anxiety, such that patients with preexisting anxiety disorders felt less affected by their own symptoms and therefore underestimated the need for treatment.

### Limitations and strengths

While large numbers of outpatient treatments (*n* = 1646) and of treatment cases involving anxiety disorders (*n* = 653) were included in the analysis, the number of cases with generalized anxiety disorder (*n* = 109) was relatively low in relation to the other subgroups (panic disorder: *n* = 294, phobic disorders: *n* = 250, no anxiety disorders: *n* = 993). The relatively small number of this subgroup made it statistically more difficult to detect differences between cases involving generalized anxiety disorder and the other subgroups. This was especially the case regarding the (uncorrected) findings of impaired self-regulation in the group involving generalized anxiety disorder compared to the group with no anxiety disorders, which did not persist in the group comparisons after applying post-hoc correction. A further limitation is the lack of a control group of healthy participants in our sample.

Due to the choice of the sample among psychotherapy outpatients, limitations of the study due to possible selection bias cannot be completely excluded. The sample was selected among psychotherapy outpatients because this group of patients included a spectrum of typical mental disorders with high prevalence in the general population (e.g., affective disorders, stress-related disorders, somatoform disorders) and also comprised a large subgroup with diagnosed anxiety disorders, which was the main target group of the present study. However, the generalisability of our findings might be limited by the fact that some patients not taking part in the study might have rejected psychotherapy as a treatment of choice and preferred exclusively psychopharmacological treatment. For instance, patients with anxiety disorders preferring to generally avoid psychotherapy might exhibit different levels of structural personality integration and also report different forms and amounts of negative and protective childhood experiences, which would not have been captured by the design of our study. The selection of patients as candidates for psychotherapy rather than exclusive psychopharmacological treatment may also have contributed to a potential selection bias, as structural integration of personality and childhood experiences may have been different in patients taking part in the study compared to those not eligible for an outpatient psychotherapy program.

However, a strength of our study is the large size of our sample, allowing for statistical detection of differences, not only between the two main groups (anxiety disorders vs. no anxiety disorders), but also on the subgroup level (generalized anxiety disorder vs. panic disorder vs. phobic disorders). Also, the amount of SCID-II diagnoses was only moderate (20% of all treatment cases), which makes potential interactions between comorbid personality disorders and group levels of structural personality integration less likely. Another strength of our study is the inclusion of a wide range of adverse childhood experiences and also protective childhood experiences in our assessment, allowing for detailed disorder-specific profiles of negative and positive early life events.

## Conclusions

Within a sample of 1646 psychotherapy outpatient treatments at the beginning of psychotherapy, we investigated differences in structural integration of personality and adverse and protective childhood experiences in patients with anxiety disorders compared to patients with no anxiety disorders, as well as between different types of anxiety disorders. Our findings underline the importance of considering structural integration of personality and childhood experiences during treatment of anxiety disorders. In particular, differential aspects of structural personality integration, as well as different kinds of childhood experiences, might be useful to address during treatment of different anxiety disorder types, for instance, aspects of self-regulation and severe forms of childhood traumatization during treatment of patients with generalized anxiety disorder.

## Electronic supplementary material

Below is the link to the electronic supplementary material.

Supplementary Material 1

## Data Availability

The data that support the findings of this study were collected at the outpatient training clinic for psychodynamic therapy at the University Hospital Heidelberg, Germany (https://www.klinikum.uni-heidelberg.de/zentrum-fuer-psychosoziale-medizin-zpm/hip/heidelberger-institut-fuer-psychotherapie-hip) but restrictions apply to the availability of these data, which are not publicly available and cannot be shared due to restrictions by the ethical review board. Data can however be requested from the author J. Nowak (jonathan.nowak@med.uni-heidelberg.de) upon reasonable request and with permission of the Ethics Committee of the Heidelberg University. The analysis code of our data analysis is publicly available at: 10.11588/data/W63LJJ.
