# Supplementary material for: Characterization of different types of anxiety disorders in relation to structural integration of personality and adverse and protective childhood experiences in psychotherapy outpatients – a cross-sectional study
Source: BMC Psychiatry. 2023 Jul 12;23:501. doi: 10.1186/s12888-023-04988-2 (PMC10339566; doi:10.1186/s12888-023-04988-2)
Supplement: Supplementary file 1 — Supplementary Material 1 [file 12888_2023_4988_MOESM1_ESM.docx]

Additional Table 1. Sample Characteristics Before and After the Start of the First Lockdown Measures in Germany

|  | Total Sample (*SD*) | Pre-Pandemic (*SD*) | During Pandemic (*SD*) |
| --- | --- | --- | --- |
| Sample Size (*n*) | 1646 | 1389 | 256 |
| Gender (Female) | 64.4 % | 63.6 % | 68.2 % |
| Age (Years) | 34.8 (13.1) | 35.1 (13.0) | 33.3 (13.5) |
| Age Range | 18 – 85 | 18 - 76 | 18 – 85 |
| Depression | 79.2 % | 80.0 % | 74.0 % |
| Anxiety Disorder | 46.5 % | 49.0 % | 31.2 % |
| Personality Disorder | 20.0 % | 21.5 % | 10.7 % |
| Substance Abuse | 13.3 % | 14.9 % | 3.3 % |
| Medication (Past) | 24.2 % | 25.8 % | 15.6 % |
| Medication (Curr.) | 21.7% | 22.03 % | 20.3 % |
| Adverse CE | 0.81 (0.61) | 0.81 (0.61) | 0.81 (0.61) |
| Protective CE | 2.52 (0.92) | 2.50 (0.9) | 2.64 (0.90) |
| OPD-SQ Mean | 1.62 (0.53) | 1.63 (0.53) | 1.57 (0.54) |

Note. Pre-Pandemic = treatment cases with intake interview before March 22, 2020 (= start of the first lockdown measures in Germany). During Pandemic = treatment cases with intake interview after March 22, 2020. Medication (Past) = psychopharmacological medication during past medical history. Medication (Curr.) = current psychopharmacological medication. Adverse CE = adverse childhood experiences, Protective CE = protective childhood experiences, OPD-SQ = Operationalized Psychodynamic Diagnosis Structure Questionnaire.
